# Phosphorylation of Glutamine Synthetase on Threonine 301 Contributes to Its Inactivation During Epilepsy

**DOI:** 10.3389/fnmol.2019.00120

**Published:** 2019-05-21

**Authors:** Deborah Huyghe, Andrew R. Denninger, Caroline M. Voss, Pernille Frank, Ning Gao, Nicholas Brandon, Helle S. Waagepetersen, Andrew D. Ferguson, Menelas Pangalos, Peter Doig, Stephen J. Moss

**Affiliations:** ^1^Department of Neuroscience, Tufts University School of Medicine, Boston, MA, United States; ^2^Mechanistic Biology & Profiling, Discovery Sciences, IMED Biotech Unit, AstraZeneca, Boston, MA, United States; ^3^Department of Drug Design and Pharmacology, Faculty of Health and Medical Sciences, University of Copenhagen, Copenhagen, Denmark; ^4^Neuroscience, IMED Biotech Unit, AstraZeneca, Boston, MA, United States; ^5^AstraZeneca Tufts Laboratory for Basic and Translational Neuroscience, Boston, MA, United States; ^6^Structure & Biophysics, Discovery Sciences, IMED Biotech Unit, AstraZeneca, Boston, MA, United States; ^7^IMED Biotech Unit, AstraZeneca, Cambridge, United Kingdom; ^8^Department of Neuroscience, Physiology and Pharmacology, University College, London, United Kingdom

**Keywords:** glutamine synthetase, phosphorylation, epilepsy, astrocyte, cAMP-dependent protein kinase

## Abstract

The astrocyte-specific enzyme glutamine synthetase (GS), which catalyzes the amidation of glutamate to glutamine, plays an essential role in supporting neurotransmission and in limiting NH_4_^+^ toxicity. Accordingly, deficits in GS activity contribute to epilepsy and neurodegeneration. Despite its central role in brain physiology, the mechanisms that regulate GS activity are poorly defined. Here, we demonstrate that GS is directly phosphorylated on threonine residue 301 (T301) within the enzyme’s active site by cAMP-dependent protein kinase (PKA). Phosphorylation of T301 leads to a dramatic decrease in glutamine synthesis. Enhanced T301 phosphorylation was evident in a mouse model of epilepsy, which may contribute to the decreased GS activity seen during this trauma. Thus, our results highlight a novel molecular mechanism that determines GS activity under both normal and pathological conditions.

## Introduction

The glutamate/GABA-glutamine cycle plays a fundamental role in the central nervous system (CNS) by detoxifying the brain of excess neurotransmitters and ammonia as well as supplying neurons with glutamine, which is a precursor of both GABA and glutamate. Glutamine synthetase (GS) catalyzes an essential step in this pathway, the ATP-dependent condensation of glutamate and ammonia into glutamine (Schousboe et al., 2013).

GS is expressed in several organs; however, its expression in the CNS is mainly restricted to astrocytes (Suárez et al., 2002). Previous studies have shown that GS is essential to survival, both in rodents and in humans (Häberle et al., 2005; He et al., 2010). Moreover, inhibition of GS activity *in vivo* induces seizures in animal models (Rowe and Meister, 1970; Eid et al., 2008; Boissonnet et al., 2012). Consistent with its central role in neurotransmitter recycling and neurotransmission, GS expression and/or activity are dramatically decreased in several neurological disorders, including mesial temporal lobe epilepsy (MTLE; Eid et al., 2004; van der Hel et al., 2005). Moreover, a recent study has demonstrated that conditional ablation of GS expression selectively in the mouse cortex of mice induces spontaneous seizures (Zhou et al., 2018).

MTLE is a recurrent, complex pathology characterized by neuronal loss and astrogliosis in the hippocampus and is the most common type of drug-resistant epilepsy (Asadi-Pooya et al., 2017). Several studies have shown that the cycling of glutamate to glutamine is slower and glutamate levels are increased in MTLE patients (Petroff et al., 2002; Cavus et al., 2005, 2008). Consistent with these findings, GS activity is significantly reduced in the sclerotic hippocampus (Eid et al., 2004; van der Hel et al., 2005), leading to the hypothesis that the loss of the enzyme’s function might contribute to the pathological state of this disorder. However, the molecular mechanisms regulating the activity of GS within astrocytes remain to be explored.

Here, we report that cAMP-dependent protein kinase (PKA) phosphorylates GS on both threonine 301 (T301) and serine 343 (S343). Additionally, we show that the main site of phosphorylation, T301, plays a fundamental role in regulating the enzyme’s activity and is significantly increased in a mouse model of epilepsy. Taken together, these results suggest that PKA-dependent phosphorylation of T301 regulates the activity of GS. Moreover, enhanced phosphorylation of this residue may contribute to the deficits in GS activity arising during epilepsy.

## Materials and Methods

### Animals

Eight to 12-week-old C57BL/6 male mice were used for phospho-specific antibody characterization and kainate injections. All mice were housed in a 12-h light/dark cycle. All mice were bred in-house at the Tufts University School of Medicine and handled according to protocols approved by the Institutional Animal Care and Use Committee (IACUC).

### Antibodies and Expression Constructs

The following antibodies were used for Western-blotting: GS mouse antibodies (Millipore, cat number MAB302 and Santa Cruz, cat number sc-398034), tubulin mouse antibody, pT301 and pS343 antibodies (created by PhosphoSolutions). GS rabbit antibody (Sigma, cat number G2781) was used for immunoprecipitation. GS was cloned into prK5 from mouse genome (ATTG) and a MYC tag was inserted between amino acids 372–373. A mutagenesis kit was used to substitute T301 and S343 into alanine (T>A: 5′AACGCCCGGCGTCTGGCTGGATTCCACGAAACC, S>A: 5′ GAAGACCGTCGGCCTGCTGCCAATTGTGACCCC).

### Cell Culture and Transfection

COS-7 cells were maintained in Dulbecco’s modified Eagle’s medium/F12 (1:1) nutrient mix with 10% fetal bovine serum and 1% of PenStrep (Thermo Fisher, cat number 11330–057). The cells were transfected by electroporation (3 μg of DNA/condition) and used 24 h after transfection. Astrocytes were prepared from forebrain of 1–4 day-old mouse pups of either sex as previously described (Schildge et al., 2013). Cells were grown in Advanced modified Eagle’s medium supplemented with 10% FBS and 1% P/S (Thermo Fisher, cat number 11995073). Confluent cultures were shaken to obtain an enriched astrocytes culture. The cells were trypsinized once a week and used 3 weeks after shaking.

### Western-Blotting

Cells or brain tissues were sonicated in lysis buffer consisting of Tris, pH 8 20 mM, NaCl 150 mM, Triton 1%, EDTA 5 mM, NaF 10 mM, Na_3_VO_4_ 2 mM, and Na pyrophosphate 10 mM. In order to detect GS, pT301 and pS343 signal, 15, 30 and 45 μg were loaded respectively from cell in culture lysates and 15, 45 and 60 μg were loaded respectively from brain lysates. Proteins separated by SDS-PAGE (10% gel) were then transferred to nitrocellulose membranes and blocked in 6% milk in PBST for 6 h. Membranes were further incubated with the appropriate primary antibody (6% milk in PBST overnight), and after extensive washes, they were probed with HRP-conjugated secondary antibodies for 1 h. Western blots were developed using an enhanced chemiluminescence system as per the manufacturer’s instructions (Amresco). Membranes were imaged (ChemiDoc MP, Bio-Rad, Hercules, CA, USA) and analyzed using ImageJ (National Institutes of Health, Bethesda, MD, USA). For quantification, the ratio between GS, pT301 and pS343 and the reporter protein (tubulin) was first analyzed. We then normalized this value to the total GS to compare the variation of T301 and S343 phosphorylation change between each condition. In order to purify the signal of pT301 antibody, it was premixed with non-phospho peptide overnight before each use.

### Immunoprecipitation and *in vitro* PKA Assay

Cells were lysed in lysis buffer consisting of 20 mM Tris, pH 8, 150 mM NaCl, 1% Triton X-100, 5 mM EDTA, 10 mM NaF, 2 mM Na_3_VO_4_, and 10 mM Na pyrophosphate for 1 h at 4°C on a circular rotor. Lysates were precleared with rabbit IgG attached to protein G beads for 4 h at 4°C. Lysates were then incubated overnight with 50 μL of protein G sepharose and 3 μg of GS antibody. Precipitated immunocomplexes were twice washed in lysis buffer. Half of the sample was then incubated in 50 μl of 40 mM Tris-HCl, pH 7.5, 5 mM magnesium acetate with 200 μm ATP, and 0.1 μg of the catalytic subunit of PKA for 30 min at 30°C, and the other half was incubated in the same reaction buffer without ATP as negative control.

### Cloning, Expression and Purification of Mouse GS

The gene sequence encoding mouse GS (2–373, Uniprot assession #P15105) was synthesized with an N-terminal 6×His-AVI-TEV tag (MHHHHHHGLNDIFEAQKIEWHEENLYFQG) and cloned into pET-24a(+) to yield pNG189 (Blue Sky Bioservices, Worcester, MA, USA). GS mutants T301A and S343A were synthesized and cloned similarly (numbering refers to the native mouse sequence). All sequences were codon optimized for expression in *Escherichia coli*. GS mutants T301E and T301V were generated with the QuikChange II Site-Directed Mutagenesis Kit (Agilent, Santa Clara, CA, USA) using pNG189 as a template according to the manufacturer’s protocol.

For expression of GS, pNG189 was transformed into BL21 (DE3) cells (New England Biolabs, Ipswich, MA, USA). Cultures were grown in LB medium with 25 μg/mL kanamycin at 37°C with shaking until they reached an OD of 0.6, at which point they were induced by addition of isopropyl β-D-1-thiogalactopyranoside to a final concentration of 500 μM. After induction, cultures were incubated at 16°C overnight with shaking. Cells were harvested by centrifugation and stored at −20°C until further use. For protein purification, all steps were performed at 4°C. Two liters worth of cells were thawed and resuspended in lysis buffer containing 50 mM sodium phosphate pH 7.5, 500 mM NaCl, 10% glycerol, 500 μM TCEP, 10 mM imidazole, and 1× Halt Protease Inhibitor Cocktail, EDTA-Free (Thermo Scientific, Waltham, MA, USA). Cells were lysed by passage through a French press. Lysates were cleared by high-speed centrifugation and loaded onto a HisTrap HP column (GE Healthcare, Chicago, IL, USA) pre-equilibrated with 50 mM sodium phosphate pH 7.5, 500 mM NaCl, 10% glycerol, 500 μM TCEP, and 10 mM imidazole. The column was washed with the same buffer before eluting with a gradient of 1 M imidazole. Fractions containing GS were identified by SDS-PAGE, pooled, and concentrated to ~2 mL. Crude GS was then loaded onto a HiLoad 16/600 Superdex 200 pg column (GE Healthcare, Chicago, IL, USA) pre-equilibrated with 50 mM sodium phosphate pH 7.5, 500 mM NaCl, 10% glycerol, and 500 μM TCEP. GS was eluted with the same buffer at a flow rate of 1 mL/min. Fractions containing GS were identified by SDS-PAGE, pooled, supplemented with 1 mM MgCl_2_ and ATP, and concentrated. Protein concentration was determined by the Bradford method (Bio-Rad; #500–006) using bovine serum albumin as a standard (Thermo Scientific, Waltham, MA, USA; #23209). GS was stored at −80°C. GS mutants were expressed and purified similarly.

### Thermal Shift Assays

Twenty-five-microliter reactions containing 5 μM wild-type (WT) or mutant GS, 5× SyproOrange (Life Technologies, Carlsbad, CA, USA; #S6651) and varying concentrations of MgCl_2_ and ATP in 20 mM HEPES pH 7.5, 300 mM NaCl, 10% glycerol, and 500 μM TCEP were prepared in white 96-well PCR plates (ThermoFisher, Waltham, MA, USA; #AB0700/W). Plates were incubated in a Bio-Rad CFX96 thermocylcer at 25°C for 2 min then heated to 95°C at a rate of 0.2°C/5 s. Plates were read at every 0.2°C interval. Melting temperatures (*T*_m_) were determined by the maximum point of the dF/dT curve of the HEX channel.

### *In vitro* Phosphorylation Assays

Fifty-microliter reactions containing 5 μM WT or mutant GS and 2.5 μM Protein Kinase A (PKA; Sigma; #P2645) in 50 mM imidazole pH 7.5, 10 mM MgCl_2_, 100 μM EDTA, 2 mM DTT, and 1 mM ATP were prepared and incubated overnight at room temperature with or without 5 μM PKI 6-22 (Calbiochem; #539684). Kinase reactions lacking PKI were stopped by the addition of PKI. Intact mass spectrometry analysis was performed on a TripleTOF 5,600+ (AB Sciex) equipped with a Duo Spray Ion Source and a Shimadzu LC 20-AD HPLC system (Shimadzu Scientific Instruments). Samples were diluted to 1 μM in 0.1% formic acid and separated on a Poroshell 300SB-C8 75 × 2.1 mm, 5 μm column (Agilent) at 30°C with a gradient of acetonitrile (5%–95%) in 0.1% formic acid. The mass spectrometer was operated in positive ion and intact protein mode. LC-MS data were acquired in TOF MS mode for m/z+ between 600 and 2,000. Peak areas for protein species were determined following spectrum deconvolution using PeakView (version 2.2) software.

### GS Activity Assays

GS activity was measured using the γ-glutamyl hydroxamate assay (Pamiljans et al., 1962). For recombinant GS, reactions were prepared in 384-well plates (Greiner; #781101) in a final volume of 10 μL by mixing 5 μL of a concentrated substrate mix containing 0–700 mM glutamate, 40 mM ATP, and 40 mM hydroxylamine in assay buffer (40 mM imidazole pH 7.4, 20 mM MgCl_2_, 1 mM DTT, and 0.01% Triton X-100) with 5 μL of concentrated enzyme mix containing either 150 nM WT GS, 700 nM GS T301A, 700 nM GS T301E, or 200 nM GS T301V in assay buffer. Negative controls lacking glutamate were prepared in parallel. Reactions were allowed to proceed at room temperature for 1 h. Reactions were terminated by the addition of 50 μL of a stop/developer solution containing 163 mM FeCl_3_, 98 mM trichloroacetic acid, and 202 mM HCl. Stopped reactions were mixed thoroughly, and their absorbances were read at 540 nm. Background absorbance from negative controls lacking glutamate was subtracted from all measurements. These data were compared to standard curves containing genuine γ-glutamyl hydroxamate (Sigma; #G2253), which were prepared in assay buffer and developed as above; kinetic parameters were extracted from non-linear fits of these data.

Assays in tissue lysates were performed as above with slight modifications. Samples were lysed by sonication in assay buffer supplemented with 1% Triton X-100, 150 mM NaCl, 25 mM β-glycerophosphate, 2.5 mM sodium orthovanadate, and 1× Protease Inhibitor Cocktail (Sigma; #P1860) and centrifuged to remove insoluble material. Several common protease and phosphatase inhibitors, including sodium fluoride, sodium pyrophosphate, phenylmethylsulfonyl fluoride, and 4-(2-aminoethyl) benzenesulfonyl fluoride were found to inhibit GS (not shown) and should be avoided. Reactions were prepared in 200 μL PCR tubes in a final volume of 20 μL by mixing 10 μL of cleared lysate and 10 μL of a concentrated substrate mix containing 700 mM glutamate, 40 mM ATP, and 40 mM hydroxylamine in assay buffer. Negative controls lacking glutamate were prepared in parallel. Reactions were allowed to proceed at room temperature for 30, 40, 50, or 60 min to ensure linearity. Reactions were terminated by adding 100 μL of stop/developer solution, after which they were centrifuged to remove insoluble material. One-hundred microliters of each supernatant was transferred to the well of a 384-well plate, and their absorbances were read at 540 nm. Background absorbance from negative controls lacking glutamate was subtracted from all measurements. Activity in lysates was compared to standard curves containing genuine γ-glutamyl hydroxamate as above and normalized by protein concentration using the Bradford method.

### PKA Inhibition Assay

The effect of methionine sulfoximine (MSO; Sigma; #M5379) on PKA (EMD Millipore; #539481) activity was measured using the PKA Colorimetric Activity Kit (Invitrogen; #EIAPKA) according to the manufacturer’s protocol.

### Data Analysis

Data were analyzed using GraphPad PRISM, and statistical significance was determined at *p* < 0.05 using one-way ANOVA followed by Dunnett’s multiple comparison *post hoc* test or Student’s *t*-test for two groups.

## Results

### GS Is Phosphorylated by PKA *in vitro*

**Figure 1:**
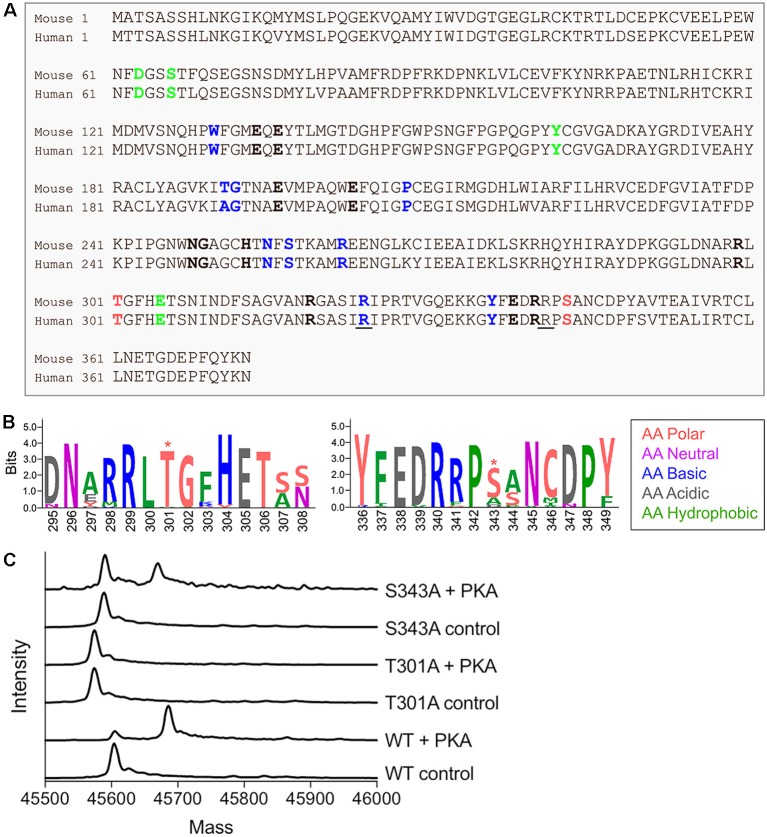
Glutamine synthetase (GS) is phosphorylated by protein kinase A (PKA) *in vitro*. **(A)** Sequence alignment between mouse and human GS protein sequences. The amino acids that bind ammonia, ATP and glutamate are respectively labeled in bold green, blue and black. The underlined amino acids highlight the reported lethal mutations in human and the red amino acids indicate the two consensus sites for PKA-dependent phosphorylation. **(B)** The weblogo motif for an alignment between bacteria (*Escherichia coli*), yeast (*Saccharomyces cerevisiae*), plant (*Arabidopsis thaliana*), worm (*Caenorhabditis elegans*), chicken (*Gallus gallus*), dog (*Canis lupus*), mouse (*Mus musculus*) and human (http://weblogo.berkeley.edu/, *highlights the putative PKA-dependent phosphorylation sites). **(C)** Representative spectra from intact mass spectrometry analysis of purified WT and mutant GS treated with PKA in the presence (control) or absence (+ PKA) of PKI. A single phosphorylation event is indicated by an 80-Da increase in mass in “+ PKA” samples relative to controls.

In mammals, GS is a homomeric decamer comprising two stacked pentameric rings that catalyzes the ATP-dependent condensation of ammonia and glutamate to glutamine (Krajewski et al., 2008). Protein sequence comparison between rodents and human shows a high percentage of similarity (98%, Figure 1A). Interestingly, both species exhibit two consensus sites for PKA-dependent phosphorylation near the active site of the protein localized on threonine 301 and serine 343 (T301 and S343, Figure 1A). However, neither of these predicted sites of phosphorylation are found in prokaryotic forms of GS (Figure 1B), suggesting that phosphorylation may play a species-specific role in controlling enzyme activity.

In order to assess the possible role that phosphorylation plays in determining GS activity, we initially expressed WT and mutant versions of mouse GS modified with an N-terminal 6× His-Avi-TEV tag in *E. coli*. WT GS and mutants in which T301 and S343 were mutated to alanine (T301A and S343A) were affinity purified and exposed to the catalytic subunit of PKA in the presence or absence of a peptide inhibitor of PKA (PKI). Intact mass spectrometry analyses revealed that WT GS and GS S343A were substrates for PKA-dependent phosphorylation (Figure 1C), as indicated by an increase in mass consistent with the addition of a single phosphoryl group in the absence of PKI. Mutating T301 to alanine completely abolished GS phosphorylation, thus indicating that T301 is the primary phosphorylation site of PKA-mediated phosphorylation of GS *in vitro*.

### PKA Phosphorylation Decreases GS Activity

**Figure 2:**
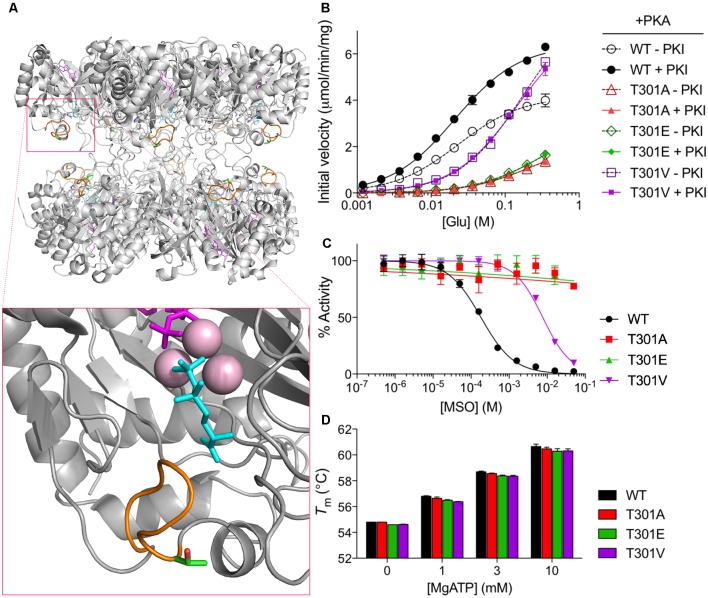
PKA phosphorylation decreases GS activity. **(A)** Overall structure of a mammalian GS decamer, with an expanded view of the active site of GS (inset) showing the locations of the glutamate flap (T301–N308; orange), T301 (carbon in green; oxygen in red), methionine sulfoximine (MSO; cyan) in the glutamate binding site, manganese ions (pink), and ADP (magenta). Based on PDB ID 2QC8. **(B)** Initial velocity vs. glutamate concentration of purified WT and mutant GS treated with PKA in the presence or absence of PKI as measured by the γ-glutamyl hydroxamate assay. Graph shows the mean ± standard error of the mean (SEM; *n* = 3). **(C)** Activity of purified WT and mutant GS pretreated with MSO for 1 h at the indicated concentrations (with 20 mM ATP in assay buffer) as measured by the γ-glutamyl hydroxamate assay. Results are normalized to the maximum activity for each protein. Graph shows the mean ± SEM (*n* = 3–5). **(D)** Melting temperatures of purified WT and mutant GS in the presence of various concentrations of MgATP as measured by differential scanning fluorimetry. Graph shows the mean ± SEM (*n* = 3).

T301 is located within the “glutamate flap,” a flexible active site loop that enhances the binding of both glutamate and ammonia and is thought to shield the active site from water, thereby preventing premature hydrolysis of reactive intermediates (Figure 2A, Supplementary Figure S1; Liaw and Eisenberg, 1994; Alibhai and Villafranca, 1994; Gill and Eisenberg, 2001; Gill et al., 2002; Krajewski et al., 2008). Previous mutational studies of GS from *Bacillus subtilis* have demonstrated that mutations in the flap can dramatically affect the enzymatic activity of GS (Fisher et al., 2002; Wray and Fisher, 2010). In order to understand how phosphorylation of T301 affects GS activity, we treated GS with PKA in the presence or absence of PKI and measured the initial velocities of γ-glutamyl hydroxamate formation at various concentrations of glutamate (Pamiljans et al., 1962). We found that unphosphorylated GS (+PKI) was highly active and displayed a low affinity for glutamate (*K*_m app_ = 21 ± 1 mM; *n* = 3; Figure 2B), which is consistent with previous studies on GS from other rodents (Richterich-Van Baerle et al., 1957; Wu, 1963; Lund, 1970). Importantly, treatment of GS with PKA in the absence of PKI decreased the specific activity of GS by approximately 40% at the highest glutamate concentration tested but did not significantly alter glutamate affinity. The decrease in activity correlated with a 74 ± 7% (*n* = 3) degree of phosphorylation of GS as measured by intact mass spectrometry (Figure 1B).

### PKA Mediates Its Effects on GS Activity *via* T301

To determine if the effects of PKA on GS activity are mediated *via* direct phosphorylation we used site-directed mutagenesis. First, we mutated T301 to glutamate (T301E), whose negative charge mimics that of a phosphoryl group. We then tested the activity of GS T301E and found, as expected, that it had very low activity (about 25% of that of WT at maximum glutamate concentration; Figure 2B) and was insensitive to PKA-treatment.

To further explore structure-function relationships at position 301, we tested the activity of GS T301A, which we expected to exhibit WT-like activity but lack the capacity for regulation by phosphorylation. GS T301A was insensitive to PKA-treatment; however, its activity was similar to GS T301E (approximately 20% of that of WT, Figure 2B). In light of the low activity observed for GS T301A, we questioned whether WT-like activity would be maintained by the introduction of a bulkier valine residue, which is found at this position in several bacterial species (Krajewski et al., 2008; Wray and Fisher, 2010). Therefore, we generated a T301V mutant and tested its activity. GS T301V exhibited a similar specific activity to WT GS but displayed a significantly lower affinity for glutamate (*K*_m app_ = 145 ± 12 mM, Figure 2B). As expected, PKA-treatment did not affect T301V activity.

In addition to alterations in glutamate-binding and activity, mutations within the glutamate flap have been shown to reduce the sensitivity of GS to the inhibitor MSO (Wray and Fisher, 2010). MSO binds to the glutamate binding site of GS in an initially reversible manner but is rapidly phosphorylated by GS in the presence of ATP; in WT GS, the glutamate flap forms strong hydrogen bonds with MSO phosphate, resulting in non-covalent but nearly irreversible inhibition (Eisenberg et al., 2000; Gill and Eisenberg, 2001; Jeitner and Cooper, 2014). We found that the IC_50_ of MSO for WT GS was 182 ± 14 μM (Figure 2C). The IC_50_ of MSO for GS T301V was 40-fold higher (7.6 ± 0.5 mM) and >100 mM for GS T301E and GS T301A.

In order to rule out the possibility that the observed differences in GS activity resulted from changes in GS stability or Mg- or ATP-binding, we performed thermal shift assays on GS in the presence of varying concentrations of MgATP, which has been shown to stabilize GS (Krajewski et al., 2008). We found that the melting temperatures of all GS constructs used in this study were similar (approximately 55°C) and that incubation with MgATP increased their melting temperatures to similar extents, indicating that the respective mutations do not impact on enzyme stability or ability to bind MgATP (Figure 2D). Collectively, these results indicate that residue T301 is essential for efficient catalysis and that alterations at this site, either by phosphorylation or mutation, can be a strong negative regulator of GS activity. Additionally, these findings are consistent with the idea that T301 phosphorylation exerts its effects on GS activity by perturbing the position or dynamics of the glutamate flap.

### Analyses of GS Phosphorylation in Cell Lines and the Brain Using Mass Spectrometry

**Figure 3:**
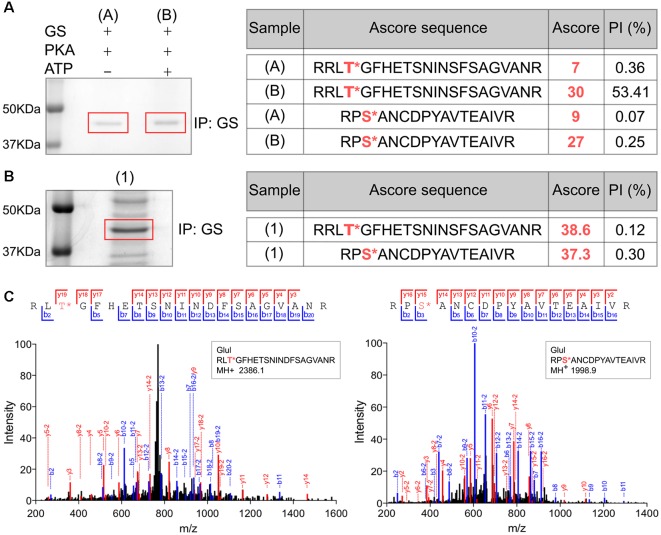
Analyses of GS phosphorylation in cell lines and the brain using mass spectrometry. **(A)** Lysates from COS-7 cells transiently transfected with WT GS were used to immunoprecipitate GS followed by an *in vitro* PKA assay. The sample (A) is the negative control (no ATP) and (B) is the experimental condition in the presence of purified PKA and 200 μM ATP (*n* = 1). The samples were analyzed by LC/MS/MS and the associated table shows the ambiguity score for each peptide (Ascore) the ratio between the peak intensity between phospho and non-phospho peptide (PI). The potential phosphorylation sites are labeled in red. **(B)** Lysates from WT mice brain (1) were used to immnoprecipitate GS (*n* = 1). The samples were analyzed by LC/MS/MS and the associated table shows the results. **(C)** Representative LC/MS/MS spectra associated for two phosphopeptides identified from GS immunoprecipitated from brain samples.

In order to assess the significance of our measurements using purified GS, we expressed GS in COS-7 cells (Huyghe et al., 2014). GS was immunoprecipitated from transfected COS-7 cells and incubated with active PKA in the presence or absence of ATP. The samples were then subjected to SDS-PAGE and stained with Coomassie blue. The major band at 45 kDa was then digested with trypsin and phosphorylation was examined using liquid chromatography coupled with mass spectroscopy (LC-MS/MS). Possible sites of phosphorylation were then ranked according to their ambiguity score (Ascore; Beausoleil et al., 2006). Both T301 and S343 exhibited Ascores >19 compared to control (-ATP), suggesting both residues are substrates for PKA-dependent phosphorylation (Figure 3A). Encouraged by our experiments using “back-phosphorylation,” we assessed phosphorylation of GS in the brain. To do so, GS was immunoprecipitated from mice hippocampal lysates. Following SDS-PAGE and Coomassie blue staining, the principal band at 45 kDa was subjected to LC-MS/MS as detailed above. Ascores >19 were seen for both T301 and S343 (Figures 3B,C). Thus, our experiments in COS-7 cells and brain reveal that in common with our experiments *in vitro*, T301 is phosphorylated by PKA. In addition, these findings suggest that GS is also phosphorylated on residue S343 in cell lines and in the brain.

### Characterization of Phospho-specific Antibodies Against GS

The results obtained using *in vitro* measurements of GS phosphorylation and our LC-MS/MS studies prompted us to create phospho-specific antibodies against T301 and S343 (pT301 and pS343, respectively). Accordingly, rabbits were immunized with a highly purified (>95%) synthetic peptide centered and chemically phosphorylated on the residue corresponding to T301 (bold) in GS: KGGLDNARRL**T**GFHETSNIND. Antibodies were raised against S343 by injecting mice with synthetic peptide centered and chemically phosphorylated on the residue corresponding to S343 (bold): KKGYFEDRRP**S**ANCDPYAVTE. High titer antisera were then subject to tandem affinity purification on the phospho- and dephospho-antigens (Jovanovic et al., 2004; Saliba et al., 2012).

**Figure 4:**
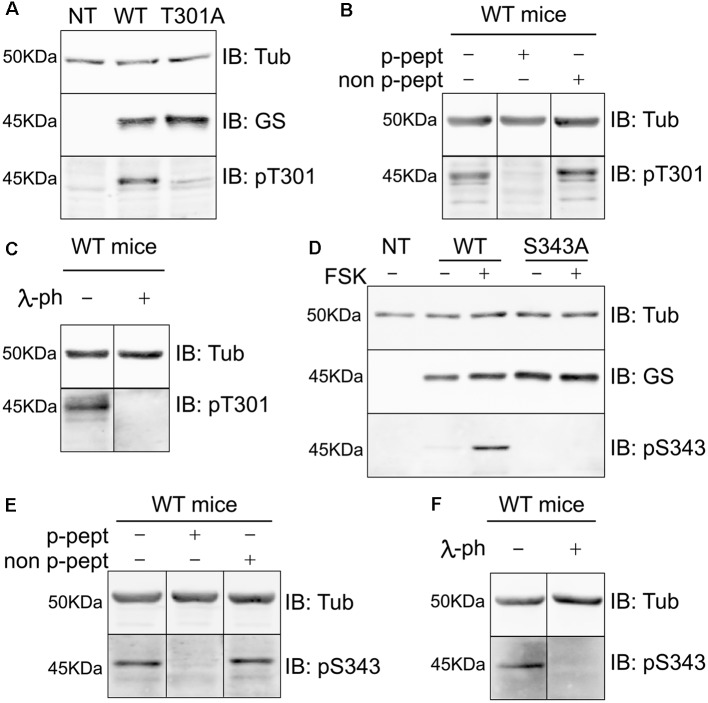
Characterization of phospho-specific antibodies against GS. **(A)** Characterization of pT301 antibody using lysates from COS-7 cells transiently transfected with GS WT or T301A point mutant. Total lysates were immnunoblotted with tubulin, GS or pT301 antibodies. Lysate from non-transfected COS-7 cells was used as an additional negative control (NT, *n* = 4). **(B)** Lysates from WT mice forebrain were used for peptide competition assays to test pT301 specificity in brain. Total lysates were immunoblotted with tubulin and pT301 antibodies (*n* = 3). **(C)** Lysates from WT mice forebrains were used for lambda-phosphatase assays and immunoblotted with tubulin and pT301 antibodies (*n* = 3). **(D)** Characterization of pS343 antibody using lysates from COS-7 cells transiently transfected with GS WT or S343A point mutant and immunoblotted with tubulin, GS or pS343 antibodies. PKA activator forskolin (FSK, 20 μM for 15 min) was used to increase S343 phosphorylation signal. Total lysate from non-transfected COS-7 cells was used as an additional negative control (NT, *n* = 3). **(E)** Lysates from WT mice forebrain were used for peptide competition assays to test pS343 specificity in brain. Total lysates were immunoblotted with tubulin and pS343 antibodies (*n* = 3). **(F)** Lysates from WT mice forebrains were used for lambda-phosphatase assays and immunoblotted with tubulin and pS343 antibodies (*n* = 3).

To test the specificity of these antibodies we expressed WT GS, T301A and S343A in COS-7 cells (Figures 4A,D respectively). Our laboratory previously determined that COS-7 cells do not express GS endogenously; therefore, those cells are a great system to study the specificity of GS phospho antibodies (Huyghe et al., 2014). Extracts of cells expressing the respective constructs were then immunoblotted with pT301, pS343 and GS antibodies. The pT301 antibody recognized a major band of 45 kDa in cells expressing WT GS, as well as a lighter band at the same molecular weight with GS T301A (Figure 4A, *n* = 4). We then performed a peptide competition assay by pre-mixing the antibody with the phospho or non-phospho peptide (Supplementary Figure S2). We observed that the pT301 signal disappears when pre-mixed with the phospho peptide but not with the non-phospho peptide, demonstrating that the pT301 antibody specifically recognizes the T301 phosphorylation site; however, the lighter band observed with the T301A point mutant also suggests that pT301 antibody recognizes a second epitope on the phospho peptide. Therefore, we tested whether we could increase the specificity of this antibody by adding an extra purification step consisting of pre-mixing pT301 with the non-phospho peptide. Detection of this band was prevented by incubation with the de-phospho-antigen (Supplementary Figure S2); therefore, we added this extra step of antibody purification for the rest of the study.

The same method was used to assess the specificity of the pS343 antibody. In contrast to T301, no basal phosphorylation of S343 was seen in COS-7 cells. In order to determine whether S343 phosphorylation could be detected by increasing PKA activity, the cells were treated with forskolin for 15 min (FSK; 20 μM), which resulted in the detection of a 45-kDa band in cells expressing WT GS but not GS S343A (Figure 4D). Significantly, the detection of this band was blocked by absorption with the phospho- but not the dephospho-antigen and by pretreatment with λ-phosphatase (Supplementary Figure S2).

In agreement with our studies conducted in COS-7 cells (Figures 4A,D, Supplementary Figure S2), pT301 and pS343 antibodies recognized bands of 45 kDa in brain extracts, the detection of which was prevented by peptide competition (Figures 4B,E) and λ-phosphatase treatment (Figures 4C,F). Collectively, these findings show that the antibodies directed against pT301 and pS343 recognize specifically the phosphorylated sites of GS. Moreover, these results suggest that the enzyme is phosphorylated on residues T301 and S343 in cell lines and the brain.

### GS Is Preferentially Phosphorylated on T301 in COS-7 Cells and Astrocytes

**Figure 5:**
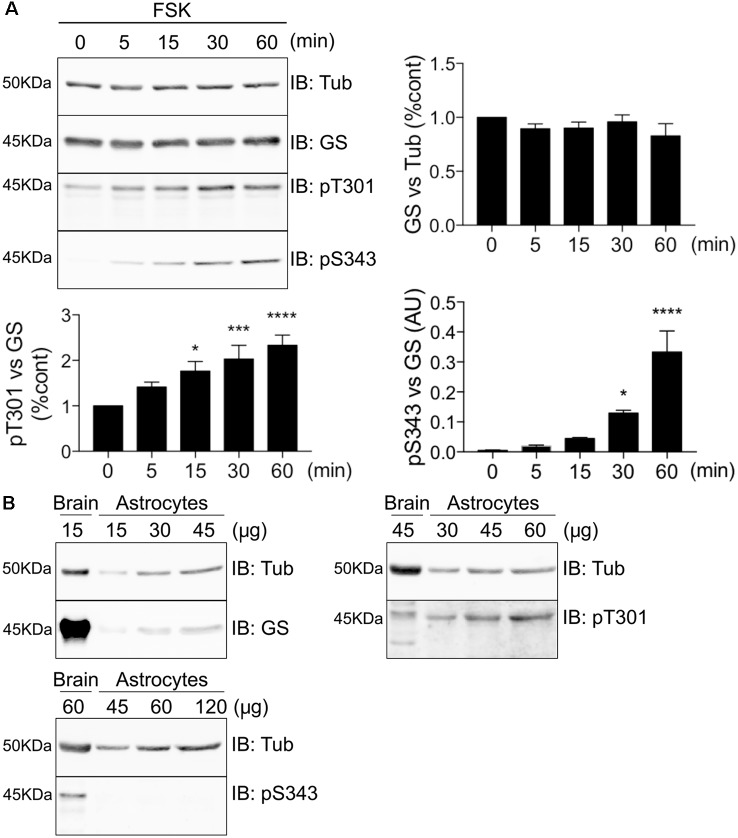
GS is preferentially phosphorylated on T301 in COS-7 cells and astrocytes. **(A)** Lysates from COS-7 cells transfected with GS WT and treated with forskolin (FSK, 20 μM) for different time-point were analyzed by immunoblotting with tubulin, GS, pT301 and pS343 antibodies. Graphs show the mean ± SEM (*p < 0.05, ***p < 0.001, ****p < 0.0001, ANOVA Dunnett’s multiple comparison, *n* = 4). **(B)** Basal phosphorylation of pT301 and pS343 in cultured astrocytes was determined by running an increasing range of protein concentration followed by immunoblotting with tubulin, GS, pT301 and pS343 antibodies. Total forebrain lysate was used as an additional control (*n* = 3).

To further assess GS phosphorylation, we expressed GS in COS-7 cells and compared the effects of FSK treatment on the phosphorylation of both residues T301 and S343 over a time course of 60 min. Consistent with our initial studies, only T301 exhibited basal phosphorylation, and FSK significantly increased phosphorylation of T301 and S343 over time. Significantly, PKA-dependent phosphorylation of T301 occurs faster (Figure 5A, 15 min = 1.77 ± 0.21, *p* = 0.011, *n* = 4) than on S343 (Figure 5A, 30 min = 0.13 ± 0.009, *p* = 0.046, *n* = 4). Next, we examined the levels of basal phosphorylation for each site in cultured astrocytes. While robust phosphorylation of T301 was seen in these primary cells, S343 phosphorylation was not detected (Figure 5B, *n* = 3). Collectively, these results in cultured cells suggest that GS is preferentially phosphorylated on T301.

### MSO Binding Decreases T301 Phosphorylation

**Figure 6:**
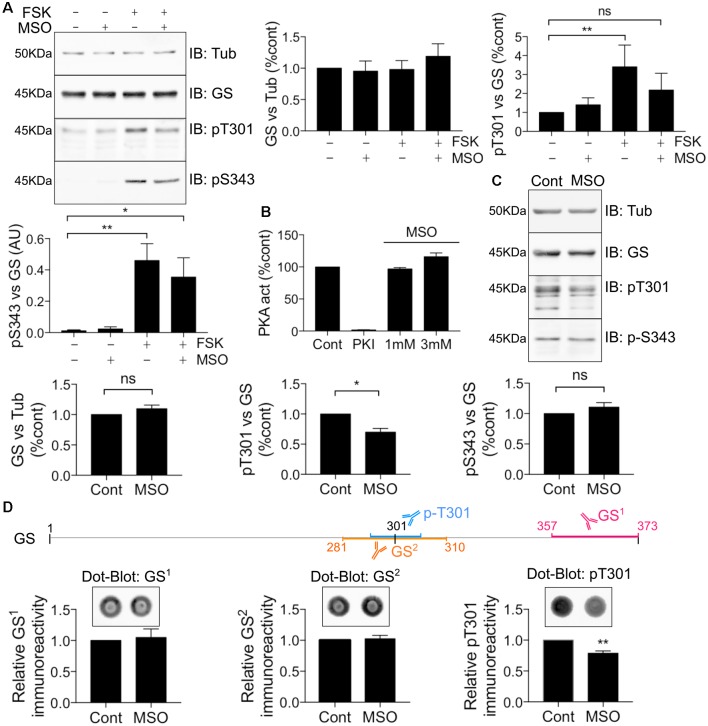
MSO binding decreases T301 phosphorylation. **(A)** COS-7 cells transiently transfected with GS WT were treated with either MSO (1 mM for 60 min) or corresponding control followed by forskolin treatment (FSK, 20 μM for 30 min). Lysates were analyzed by immunoblotting with tubulin, GS, pT301 and pS343 antibodies. Graphs show the mean ± SEM (*p < 0.05, **p < 0.01, ns, not significant, ANOVA Dunnett’s multiple comparison test, *n* = 4). **(B)** Relative activity of purified mouse PKA treated with either PKI (1 mM) or MSO as measured by an ELISA-based assay using an immobilized peptide substrate. Graph shows the mean ± SEM (*n* = 3). **(C)** WT male mice (8–12 weeks) were injected intraperitoneally by saline or MSO (75 mg/kg). Dissections of the hippocampi were performed 8 h after injection and the tissues lysates were analyzed by immunoblotting with tubulin, GS, pT301 and pS343 antibodies. Graphs show the mean ± SEM (*p < 0.05, ns, not significant, unpaired *t*-test, *n* = 5). **(D)** The schema represents the antigen region recognized by different GS antibodies. Hippocampal lysates used in **(C)** were analyzed by Dot-blot for pT301 and two different GS antibodies (GS^1^ and GS^2^). Graphs show the mean ± SEM (**p < 0 0.01, unpaired *t*-test, *n* = 5).

MSO is a well-characterized GS inhibitor that binds irreversibly to the glutamate binding site within this enzyme (Eisenberg et al., 2000; Cloix et al., 2010). Given the strategic localization of T301 within the active site, we further sought to assess whether MSO affects the ability of PKA to phosphorylate GS. To do so, COS-7 cells transfected with WT GS were exposed to 1 mM MSO for 60 min followed by 20 μM FSK for 30 min. Pre-treatment with MSO partially blocked the FSK-induced potentiation of T301 phosphorylation compared to control (Figure 6A, FSK = 3.41 ± 1.15, MSO+FSK = 2.19 ± 0.88, *n* = 4). Likewise, we observed that MSO tended to partially reduce FSK-induced potentiation of S343 phosphorylation; however, a significant increase was still observed compared to control condition (Figure 6A, FSK = 0.46 ± 0.11, MSO+FSK = 0.35 ± 0.12, *n* = 4, *p* = 0.03).

To assess the significance of the results obtained in COS-7 cells for events in the brain, mice were injected with either saline or a pro-convulsive dose of MSO (75 mg/kg, Bernard-Helary et al., 2000; Cloix et al., 2010). All MSO-injected mice started seizing between 4 and 6 h after injection. After 8 h, the mice were sacrificed and total GS levels and phosphorylation of T301 and S343 were analyzed from hippocampal lysates. MSO treatment significantly decreased T301 phosphorylation relative to vehicle-treated controls (Figure 6C; MSO = 0.70 ± 0.06, *p* = 0.0185, *n* = 5). We did not observe any significant change in S343 phosphorylation in MSO-injected mice compared to controls (Figure 6C, MSO = 1.11 ± 0.07, *n* = 5). These results suggest that MSO treatment specifically reduces phosphorylation of residue T301 in the hippocampus of WT mice. However, a previous study has shown that MSO binding impairs the antibody-antigen recognition that might lead to misinterpreting the reduction of the signal for a decrease in protein levels (Bidmon et al., 2008). To circumvent this technical issue, the hippocampal samples from the saline or MSO-injected mice were used to perform dot-blot analyses to compare their respective immunoreactivity after an additional denaturation step (10 min at boiling temperature). As an additional control, a GS antibody targeting the region surrounding the T301 residue was also used (GS^2^). We did not detect any change in GS signal using the regular GS antibody (GS^1^) or GS^2^, however, a reduction in the T301 signal was still evident (Figure 6D). Therefore, MSO binding leads to a decrease of T301 phosphorylation *in vivo*. Finally, to investigate any possible direct inhibitory effects of MSO on PKA activity, we directly tested how MSO affected the enzyme activity of PKA *in vitro*. Under these conditions, MSO did not directly modify PKA activity (Figure 6B). Accordingly, these results provide further evidence that T301 phosphorylation is intimately linked to the catalytic mechanism of GS in the hippocampus of WT mice.

### T301 Phosphorylation Is Increased and GS Activity Is Decreased in Chemico-induced *Status Epilepticus*

**Figure 7:**
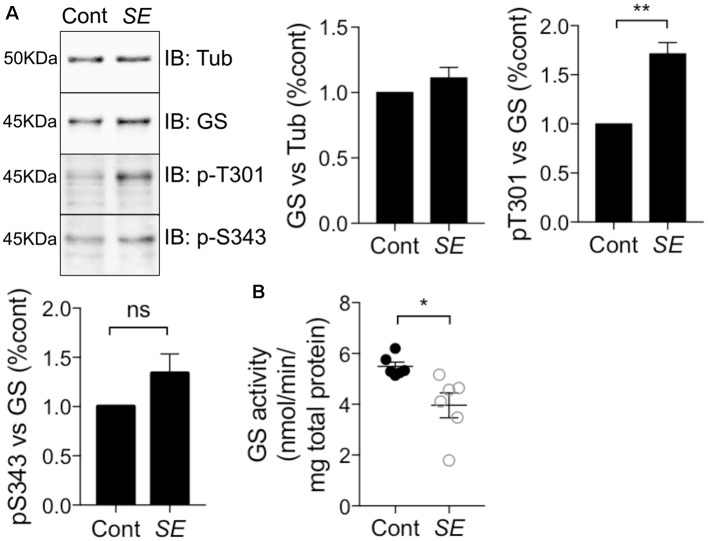
T301 phosphorylation is increased and GS activity is decreased in chemico-induced *Status Epilepticus (SE)*. **(A)** WT male mice (8–12 weeks) were injected intraperitoneally by saline or kainate (KA, 20 mg/kg). Dissections of the hippocampi were performed 1 h after SE and the tissues lysates were analyzed by immunoblotting with tubulin, GS, pT301 and pS343 antibodies. Graphs show the mean ± SEM (*p < 0.01, ns: not significant, unpaired *t*-test, *n* = 6). **(B)** GS activity in hippocampal lysates from kainate- (*SE*) or saline-injected (Cont) mice as measured by the γ-glutamyl hydroxamate assay. Graph shows the mean ± SEM (*p < 0.05, unpaired *t*-test with unequal variances, *n* = 6).

Epilepsy leads to deficits in GS activity (Eid et al., 2004; van der Hel et al., 2005); however, the underlying molecular mechanisms are ill-defined. Thus, we assessed if alterations in GS phosphorylation may be of significance in a mouse model of epilepsy. To do so, we injected mice with the chemico-convulsant kainic acid (KA; 20 mg/kg, Silayeva et al., 2015). Subsequent to KA injection, the development of *Status Epilepticus* *(SE)* was measured by the development of stage V seizures as defined on the Racine Scale (Racine et al., 1972). Sixty minutes after entrance into *SE*, mice were sacrificed and hippocampal lysates were subjected to immunoblotting with GS, pT301 and pS343 antibodies. This revealed that T301 phosphorylation was significantly increased in *SE* relative to control (Figure 7A, *SE* = 1.71 ± 0.12, *n* = 5, *p* = 0.0017). In contrast, pS343 signal (*SE* = 1.41 ± 0.14, *n* = 5, *p* = 0.27) and total GS (*SE* = 1.11 ± 0.04, *n* = 5, *p* = 0.85) were not affected in KA-treated mice. Finally, we assessed if in addition to modifying GS phosphorylation, *SE* impacts on its activity using the γ-glutamyl hydroxamate assay. The results revealed that GS activity was reduced by about 25% in KA-injected mice relative to saline-injected controls (Figure 7B, control = 5.5 ± 0.17, *SE* = 3.4 ± 0.49, *n* = 6, *p* = 0.02). Thus, increased phosphorylation of T301 may contribute to the reduced activity of GS activity observed in epilepsy.

## Discussion

In the brain, GS expression is restricted to astrocytes where it plays a critical role in regulating glutamate levels and ammonia by catalyzing their conversation to glutamine. Thus, GS is accepted to play a central role in regulating amino acid-mediated neurotransmission. Studies in peripheral tissues and the CNS suggest that GS activity is modulated by posttranslational mechanisms that include acetylation, tyrosine nitration, oxidation, and ubiquitination (Bidmon et al., 2008; Castegna et al., 2011; Nguyen et al., 2016).

GS shows remarkable phylogenic conservation, but it is notable that all mammalian isoforms contain consensus sites for phosphorylation by some protein kinases, including PKA, that are not found in prokaryotes. To assess the importance of phosphorylation—the most common protein covalent modification—in regulating GS activity, we expressed and purified the murine enzyme from *E. coli*. *In vitro*, GS is phosphorylated by PKA to a final stoichiometry of 70% solely on T301. T301 is within the glutamate flap region of GS, which plays a critical role in substrate binding and is essential for efficient catalysis (Alibhai and Villafranca, 1994; Liaw and Eisenberg, 1994; Gill and Eisenberg, 2001; Gill et al., 2002). Phosphorylation of GS significantly reduced its ability to synthesize glutamine, an effect that was critically dependent on T301, as demonstrated by mutation.

Mutation of T301 to either a glutamate residue, which mimics the negative charge of a phosphoryl group or a conservative alanine residue dramatically decreases enzyme activity and increases the apparent *K_m_* for glutamate. In prokaryotes, a hydrophobic valine residue is found at this position, and conversion of T301 to valine in murine GS results in a relatively mild reduction in glutamate affinity. In addition to these effects on the catalytic properties of GS, mutating T301 greatly reduced the affinity of GS for its inhibitor MSO. Importantly, mutations of T301 did not modify GS stability or its ability to bind ATP and magnesium, providing further evidence that these mutations selectively impact glutamate binding. Collectively these results suggest a vital role for the phosphorylation of T301 in modulating the activity of GS, consistent with its role in substrate binding (Krajewski et al., 2008; Wray and Fisher, 2010).

To better understand the significance of our *in vitro* measurements, we examined the phosphorylation of GS when expressed in COS-7 cells and the brain. Confirming our experiments with purified PKA, LC-MS/MS revealed that T301 was phosphorylated in both systems. In addition, a second site of phosphorylation, S343, was detected. In contrast to T301, S343 is buried near the monomer-monomer interface (Supplementary Figure S1), and its role remains unknown. In agreement with our LC-MS/MS measurements, several high-throughput mass spectrometry studies have detected both T301 and S343 phosphorylation in the murine brain (Goswami et al., 2012; Trinidad et al., 2012). To further study GS phosphorylation, we produced phospho-specific antibodies against T301 and S343. These tools confirmed that both residues were phosphorylated in COS-7 cells and brain lysates. Significantly, in COS-7 cells, phosphorylation of T301 was faster than S343 upon activation of PKA. In cultured astrocytes, phosphorylation of S343 was not detected under basal conditions, but robust phosphorylation of T301 was seen. Collectively, these results suggest that T301 is preferentially phosphorylated upon the activation of PKA. They further suggest that S343 phosphorylation may be regulated in a cell type- and/or context-specific manner.

To further assess the relationship between phosphorylation of T301 and GS structure, mice were injected with MSO, which irreversibly binds to the enzyme’s active site. Eight hours after MSO injection, decreased T301 phosphorylation was evident in the brain, while S343 and total GS levels were unaffected. Thus, these results suggest that phosphorylation of T301 is intimately linked with the catalytic mechanism of GS in the brain. Finally, we assessed the effects of *SE* on GS phosphorylation and activity. Deficits in GS activity and increased T301 phosphorylation were evident in mice exhibiting *SE*, while total GS levels and S343 phosphorylation were unaltered. Therefore, enhanced T301 phosphorylation may contribute to the deficits in GS activity that have been reported in human patients and animal models of epilepsy (Eid et al., 2004; van der Hel et al., 2005; Eid et al., 2012).

Collectively, our studies have revealed that GS is subject to PKA-mediated phosphorylation, which leads to its inhibition after *SE*. Thus, preventing its phospho-dependent inactivation may be a potent mechanism to upregulate GS activity, which may be of therapeutic value in epilepsy. Consistent with this notion, PKA activity has been shown to be elevated in animal models of *SE* and in human epileptic foci (Rakhade et al., 2005; Lee et al., 2007; Bracey et al., 2009). It is important to note that T301 in addition to S343 may also be subject to phosphorylation by other protein kinases such as protein kinase C. Thus, the phosphorylation of GS may be subject to modulation by multiple cell signaling pathways under control conditions and during seizures. Finally, as decreased GS activity has been implicated in schizophrenia (Steffek et al., 2008) and Alzheimer’s disease (Smith et al., 1991), alterations in its phosphorylation may also be relevant to these pathologies.

## Ethics Statement

All mice were bred in-house at the Tufts University School of Medicine and handled according to protocols approved by the Institutional Animal Care and Use Committee (IACUC).

## Author Contributions

DH performed cell culture, dissections, mutagenesis, antibody characterization, *in vivo* injection and Western blotting, and data analysis. AD performed enzyme purifications, *in vitro* phosphorylation, enzyme assays, thermal shift assays and data analysis. CV and PF performed antibody characterization, cell culture, Western-blotting. NG performed intact mass spectrometry. Experiments were designed by DH, AD, PD and SM. The manuscript was written by DH, AD and SM with input from PD, CV, NB, PF, HW, NG, AF, MP. DH and SM conceived the project.

## Conflict of Interest Statement

AD, NG, NB, PD and MP are current employees of AstraZeneca. SM acts as a consultant for AstraZeneca and SAGE Therapeutics, relationships that are governed by Tufts University. The remaining authors declare that the research was conducted in the absence of any commercial or financial relationships that could be construed as a potential.
