# Supplementary figures and images for: Phosphorylation of Glutamine Synthetase on Threonine 301 Contributes to Its Inactivation During Epilepsy
Source: Front Mol Neurosci. 2019 May 21;12:120. doi: 10.3389/fnmol.2019.00120 (PMC6536897; doi:10.3389/fnmol.2019.00120)

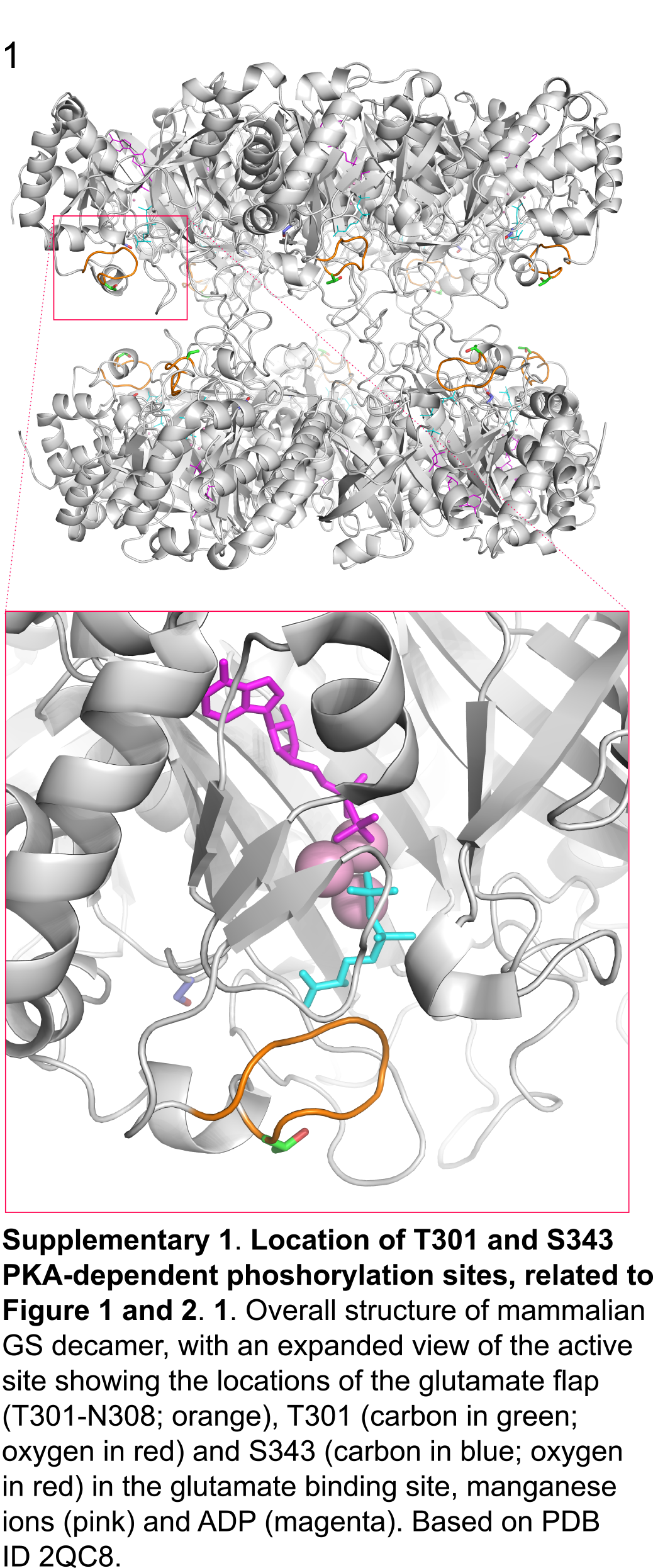

Supplement: Supplementary file 1 [file Image_1.TIF]

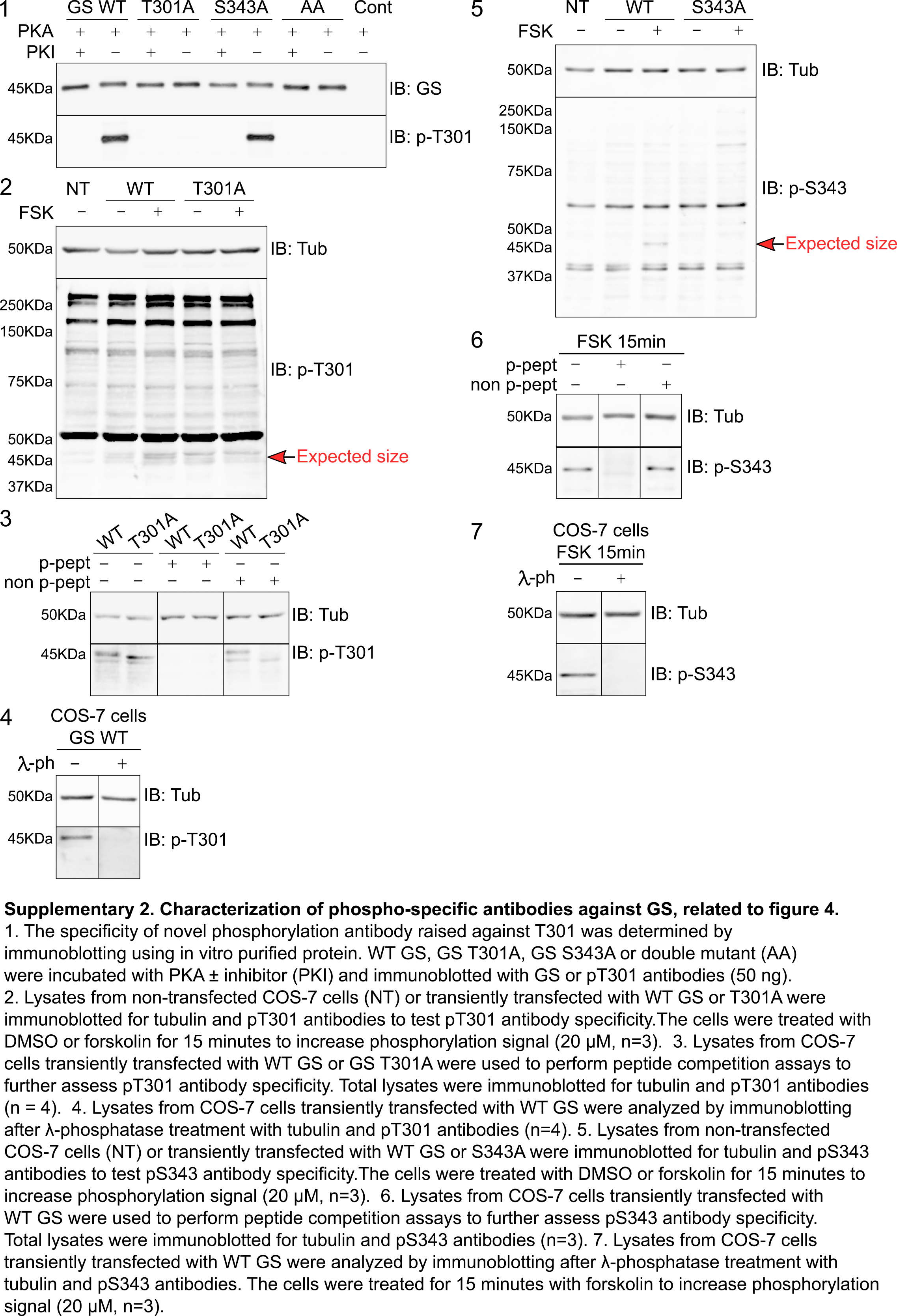

Supplement: Supplementary file 2 [file Image_2.TIF]
